# Multiple Site Fracture of Both Rods in a Malleable Penile Implant

**DOI:** 10.1155/2016/9564904

**Published:** 2016-03-15

**Authors:** Marcelo Almeida Pinheiro, Haroldo Brasil Barroso Filho, Francisco José Cabral Mesquita, Ivon Teixeira de Souza, Rafael Silva Guimarães, Everaldo Moura Santos, Rômulo Augusto da Silveira, Rommel Prata Regadas, Geraldo Munguba Macedo

**Affiliations:** ^1^School of Medicine, Unichristus University, 60190-060 Fortaleza, CE, Brazil; ^2^Urology Department, Santa Casa da Misericórdia de Fortaleza, 60025-060 Fortaleza, CE, Brazil; ^3^Sexual Medicine Department, Santa Casa da Misericórdia de Fortaleza, 60025-060 Fortaleza, CE, Brazil

## Abstract

Penile prosthesis implant is the definitive treatment for refractory erectile dysfunction. Fracture of malleable prosthesis is rarely described due to its low incidence. We describe a case of multiple, bilateral fracture of a malleable penile implant, ten years after implantation. After the diagnosis, a review surgery was performed and the implants were replaced. No corporal rupture or urethral lesion was observed. Review of the literature shows few articles reporting penile implant fractures, and to our knowledge no other article has described multiple, bilateral fractures of a penile prosthesis.

## 1. Introduction

Penile prosthesis implantation is the definitive treatment for erectile dysfunction refractory to conservative treatments, despite the existence of multiple, effective, and safe medications [1, 2].

The available types of implants are inflatable (3 pieces and 2 pieces) and malleable [3]. Success rates with malleable implants are around 92% and tend to have fewer complications compared to inflatables [4].

Malleable implants are easier to place and to use and have a lower rate of mechanical failure and lower costs. Complications may occur during or after the surgical procedure. The most common complications during implantation are corporeal and urethral perforation, and after the procedure they are haematoma formation, infection, pain, deformity, and erosion [2].

Penile prosthesis fracture is a rarely described complication due to its infrequent occurrence. To our knowledge, a multiple fraction of both rods has never been described.

## 2. Case Report

A 72-year-old white male presented to our urology service complaining of “fracture” of his penile implant. He denied having pain or voiding difficulties. He first noticed a loss of continuity on the right rod of the penile implant, after a sexual intercourse, three months prior to his consultation.

**Figure 1:**
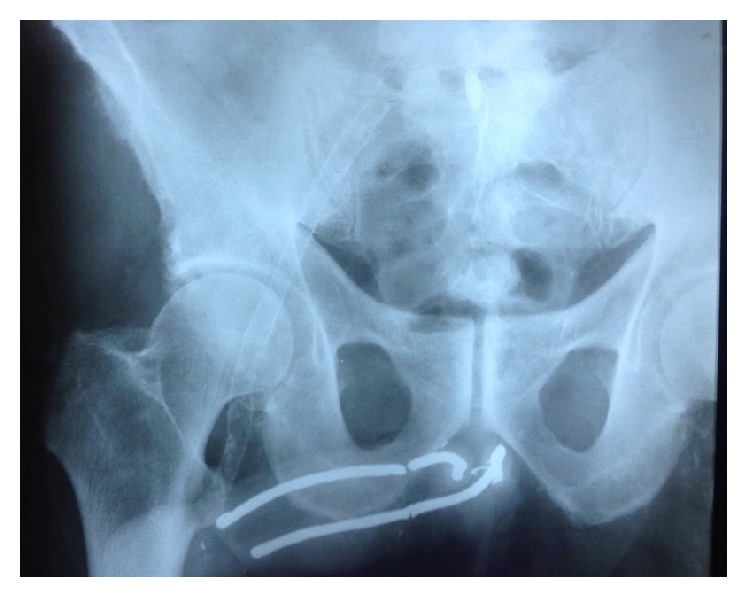
Pelvic radiography, showing total fracture of the medial portion of the right and left rods and probable fracture of the base of both rods.

At examination, a fracture of the rods was observed. There was no haematoma or signs of infection. Labs were normal except for high glycemic levels. Patient had uncontrolled type II diabetes mellitus. The patient underwent a pelvic radiography, which showed total fracture of the medial portion of the right and left rods and probable fracture of the base of both rods (Figure 1).

This penile prosthesis was implanted in a different service ten years prior, due to vascular erectile dysfunction, aggravated by a surgery for correction of a lumbar herniated disc, after which he developed fecal incontinence that improved with physiotherapy. He is a former smoker (60 packs/year) and a heavy drinker. He had an aortofemoral bypass, distal right foot, and left toe amputation due to vascular insufficiency and had congestive heart failure. He reports placing his implant in a lower resting position over the years.

**Figure 2:**
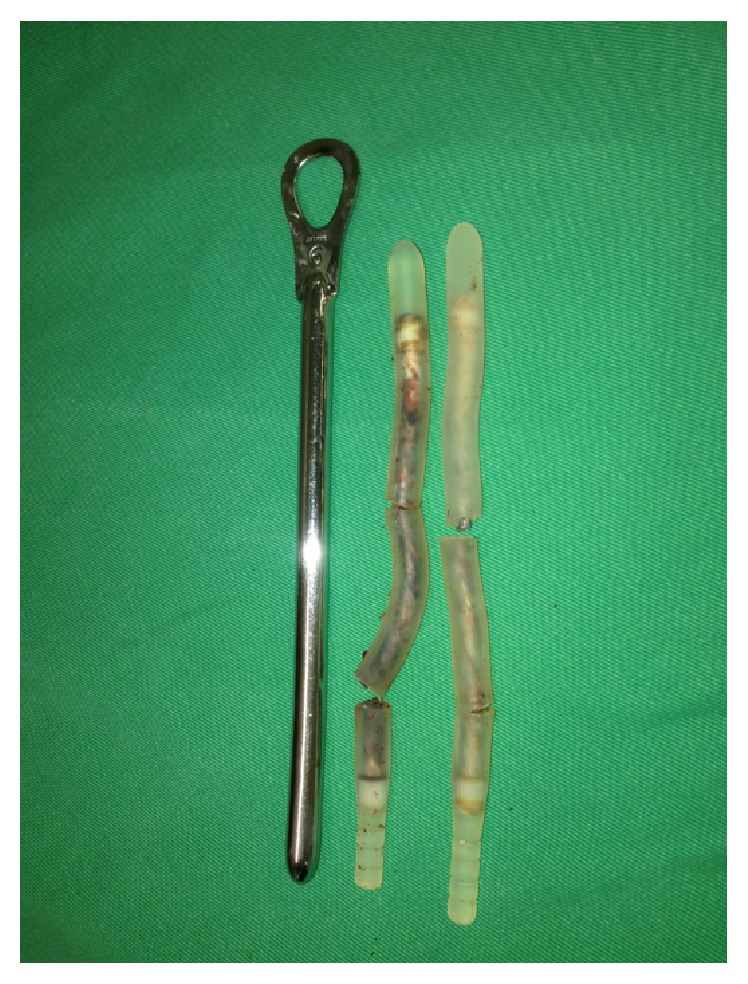
Surgical specimen after review procedure. Total fracture of the medial portion of the right and left rods. The left rod was fractured in three places.

The patient was submitted to a review surgery and the defective implant was replaced. The right rod was replaced without any trouble. The left rod was fractured in three places and the corporotomy had to be enlarged to remove the most proximal fragment (Figure 2). After extensive irrigation with a solution of Gentamicin and normal saline, the new prosthesis (9 mm, Medicone) was implanted. Patient was discharged in the second day post-op without any complaints.

He developed a small skin dehiscence without any signs of infection and was treated conservatively. At three-month follow-up appointment, the wound was healed and he reported using the implant without any trouble.

## 3. Discussion

Penile prosthesis is an effective treatment for refractory erectile dysfunction [1].

Mechanical failure is an unusual complication following malleable prosthesis implantation. A few reported cases describe this complication. Akand et al. described failure 6 years after implantation [5]. Minervini et al. in a series of 393 cases described two cases of mechanical failure after 6 and 9 years of implantation [3].

Lee et al. reported the first two cases of bilateral rod fracture after 5 and 6 years after the implant procedure [6].

Recently Bozkurt et al. reported a case of bilateral rod fracture, without compromise of the silicone external layer, and attributed this complication to a mechanical failure. The patient did not report vigorous sexual activity or trauma during the 14 years of use [7].

As in our case, there was no haematoma or lesion of the cavernous bodies in any of the described cases. Similarly, the review surgeries were performed without any major complications.

Diagnosis was made by history and physical examination. Radiology confirmed the diagnosis. In difficult cases, magnetic resonance imaging may be useful [8].

Diabetes patients have a higher probability of wound complications; however, a strict glycemic control may reduce this risk [9]. The patient described here had elevated glycemic levels and peripheral vasculopathy, which may have contributed to the wound complications.

Most of the cases reported were unilateral. Özgür et al. reported a similar case with bilateral rod fracture [10]. However, bilateral multiple rod fractures were not previously reported.

The real incidence of a penile prosthesis fracture may be underestimated due to lack of symptoms. In this case report, there was no voiding difficulty or haematoma. In a retrospective analysis, Pozza et al. reported 5 cases of a rod problem in which the patients preferred to retain only one rod, with satisfactory penetrative function. One patient preferred to maintain broken rods without changing them [11]. Paranhos et al. also described three patients who had a fracture of the prosthesis without being aware of the problem; all had normal sexual activity [12].

Patients should be counseled on situations that increase the risk of brakeage (woman on top and vigorous sexual activity) [13] and the importance of lubricant use [12].

This is the first reported case of multiple bilateral malleable penile prosthesis fracture. The malleable implant may suffer mechanical failure after prolonged usage. Review surgery was performed without major complications. Despite the low frequency, patients should be informed of the possibility of mechanical failure and how to recognize it. Patients should also be informed on the proper use of the prosthesis and factors that contribute to its fracture.
